# Distribution of bla_CTX-M_, bla_TEM_ and bla_SHV_ encoding-genes among extended-spectrum beta-lactamase (ESBL)-producing *Escherichia coli* isolates at the different hospitals in Tabuk, Kingdom of Saudi Arabia

**DOI:** 10.1371/journal.pone.0350207

**Published:** 2026-09-10

**Authors:** Ruqaiah Ismael Bedaiwi, Marah Naif Alatawi, Bernard Silvala

**Affiliations:** Department of Medical Laboratory Technology, Faculty of Applied Medical Sciences, University of Tabuk, Tabuk, Saudi Arabia; Suez Canal University, EGYPT

## Abstract

The rising incidence of *Escherichia coli* (*E. coli*) strains harbouring ESBL enzymes in healthcare environment including hospitals poses significant public health concerns. This study sought to assess the occurrence of *E. coli* strains that carry ESBL enzymes in clinical isolates from patients at hospitals in Tabuk, Saudi Arabia, focusing on the responsible genes and antibiotic susceptibility profiles. The 100 clinical samples positive for these ESBL genes were collected from the different Tabuk hospitals’ in-patients for a 6-month period only, with multiplex PCR used to identify genes that encode ESBLs (*bla*_TEM_, *bla*_SHV_, *bla*_CTX-M-group 1_, *bla*_CTX-M-group 9_, and *bla*_CTX-M-group 25_). These results yielded that 24% of the clinical isolates exhibited positive for ESBL, with the CTX-M-1 gene having the highest prevalence (67% of ESBL-positive samples). This study underscores the high occurrence of the CTX-M-1 gene isolated in ESBL (+) *E. coli* within the Tabuk region, providing critical insights into local antibiotic resistance patterns. While these findings enhance our understanding of regional dynamics, they also reaffirm existing knowledge regarding global trends in ESBL gene prevalence. Other genes detected included *bla*_SHV_ (46%), *bla*_TEM_ (50%), *bla*_CTX-M-9_ (63%), and *bla*_CTX-M-25_ (50%). Co-occurrence of *bla*_SHV_ and *bla*_CTX-M-1_ genes was observed in 42% of isolates, while *bla*_TEM_ and *bla*_SHV_ co-occurred in 12.5%. These ESBL-positive *E.coli* were primarily isolated from urine (70.8%), wound (12.5%) and blood (16.5%). While these enzymes confer resistance to many beta-lactam classes, they generally remain sensitive to carbapenems, cephamycin and certain β-lactamase inhibitors, while cefoxitin showed 95.8% resistance in this study. Although the study was conducted over a limited six-month duration with a relatively small sample size, the isolation of these specific strains is nonetheless significant. These outcomes highlight the necessity for ongoing surveillance studies and robust infection control programs to combat antibiotic resistance in healthcare settings. To address the study’s objectives, it is recommended to develop targeted antibiotic stewardship programs that align with local resistance patterns, enhance surveillance systems to monitor ESBL-producing bacteria, and implement stringent infection control protocols in healthcare settings. Additionally, launching educational initiatives for healthcare professionals and the public, promoting research on resistance mechanisms, and collaborating with public health authorities will ensure that findings inform evidence-based policies. Finally, fostering multidisciplinary approaches will help create comprehensive strategies for effectively managing antibiotic resistance at both local and global levels.

## Introduction

Extended-spectrum beta-lactamases (ESBLs) are commonly linked with *Escherichia coli*, which is the predominant pathogen responsible for uncomplicated cystitis. In addition to urinary tract infections (UTIs), *E. coli* is implicated in various extra- intestinal infections, which includes pneumonia, bacteremia, and abdominal infection such as spontaneous bacterial peritonitis. Moreover, it is a significant contributor to nosocomial infections, particularly catheter-associated UTIs and ventilator-associated pneumonia (VAP) [1].

The ability of various pathogenic strains of *E. coli* to yield ESBLs is largely associated to the horizontal transfer of ESBL genes. This genetic exchange facilitates the emergence of high-risk clones that spread within populations, contributing to the increasing prevalence of antibiotic-resistant infections [2]. The public health implications of these resistant strains are profound, necessitating ongoing surveillance and novel therapeutic strategies to combat their spread.

In Saudi Arabia, multiple research studies highlighted the rising increase of ESBL-producing *E. coli* and their unique challenges faced in the region. For instance, research has shown an alarming rate of ESBL production in clinical isolates, which poses significant risks to patient outcomes and complicates treatment protocols [3]. The limited availability of advanced diagnostic tools and effective antibiotics further exacerbates the situation, making it crucial to address these issues through targeted interventions.

A primary way these bacteria acquire antibiotic resistance is by producing enzymes called β-lactamases, which encompass extended-spectrum β-lactamases (ESBLs). These enzymes are capable of hydrolyzing and neutralizing a wide spectrum of beta-lactam antibiotics, like the penicillin and cephalosporin, as well as monobactam. While these enzymes confer resistance to many antibiotic classes, they generally remain sensitive to carbapenems, cephamycin, and certain β-lactamase inhibitors like clavulanic acid [4].

The onset of ESBL-producing bacteria can be tracked down in Western Europe, where the widespread use of expanded-spectrum β-lactam antibiotics likely facilitated their development [5]. Subsequently, ESBLs have been detected in various regions, such as in Asia and America. The occurrence of ESBL formation varies significantly across different countries, contributing to the increase of multi-drug resistant patterns in pathogenic bacteria, particularly in healthcare settings worldwide [6].

The global health implications of antimicrobial resistance (AMR) are profoundly concerning. In 2019, antimicrobial resistance was notably responsible for an estimate of 1.27 million deaths worldwide. Projections suggest that the data could escalate to 10 million fatalities every year until 2050 unless effective interventions are put in place [7].

Over the last several years, this characterization of extended-spectrum beta-lactamase (ESBL) has advanced significantly, with over 300 distinct variants identified. The majority of these ESBLs are found within the Enterobacteriaceae family, particularly in the different strains of *Klebsiella pneumoniae* and *Escherichia coli* [6]. This proliferation of ESBL-producing organisms raises serious concerns due to the increasing genetic diversity among these bacteria, which poses substantial challenges for healthcare facilities. These microorganisms can undermine the efficacy of commonly used antibiotics, thereby limiting treatment options and exacerbating the issue of antibiotic resistance in clinical settings. Consequently, there is an urgent need to comprehend the genetic determinants of resistance in ESBL-producing bacteria, as this information is significant to develop effective plans and strategies to avert and stop antibiotic resistance within healthcare environments [8].

The rise of resistance in antibiotics among Gram-negative bacteria is particularly troubling, primarily due to their outer membrane, which serves as a formidable barrier to many antibiotics, reducing their effectiveness. The mechanisms by which these bacteria acquire resistance include the action of efflux pumps, modifications of porin proteins, and the production of enzymes that can inactivate antibiotics [9]. In light of these challenges, ongoing research and targeted public health initiatives in Tabuk are essential to mitigate the impact of ESBL-producing bacteria on community and hospital settings.

Addressing these challenges is essential for protecting public health and ensuring the effectiveness of current antimicrobial therapies. Therefore, this research aimed to explore the existence of the different genes responsible for ESBL production such as *bla*_CTX-M- group 25_, *bla*_CTX-M-group 1,_ *bla*_SHV_, *bla*_TEM_ and *bla*_CTX-M- group 9_ among *E. coli* strains that produce ESBL and the pattern of drug resistance from these clinical samples across multiple hospitals in Tabuk, Kingdom of Saudi Arabia. Any findings can be utilized to inform recommendations for decision-making and public health interventions.

## Methods

### Bacterial isolates

In this study, a total of 100 (non-duplicate) clinical specimens, including blood, urine, and wound swabs, were meticulously acquired for a 6- month period from the Microbiology laboratory of three key hospitals in Tabuk (Kingdom of Saudi Arabia) —King Fahd Specialist Hospital, King Khalid Hospital, and Children and Maternal Hospital—between December 2023 and May 2024. These hospitals, recognized for their governmental and tertiary status, cater to a diverse patient population within the Tabuk province. Duplicate isolates (defined as more than one isolate of *E. coli* recovered from the same patient within a 30-day period) were excluded from the analysis to avoid overrepresentation of individual patients. All clinical samples underwent phenotypic identification and ESBL confirmatory testing using the Vitek system II. The inclusion criteria comprised only clinical isolates suspected of harboring *E.coli* with ESBL enzymes that tested positive in the Vitek system II. Patient information was not collected to uphold anonymity and maintain ethical standards, ensuring no direct patient interaction during sample collection. Ethical clearance and permission were diligently secured for this study, and stringent protocols for proper storage and handling were adhered to throughout the research process. By following these procedures and selecting hospitals based on their demographic diversity and advanced laboratory capabilities, the present study endeavors to offer a comprehensive understanding of ESBL distribution in Tabuk region.

### Research project ethical approval

Approval for the research ethics clearance was procured from the committee of the University of Tabuk- Research Ethics Committee (REC # UT-320-163-2023), dated November 29, 2023.

**Approved Letter from Ministry of Health (MOH) for the Collection of Clinical Isolates** was obtained and approved on March 17, 2024.

### Screening, Identification and Confirmation of ESBL Production

#### Identification of ESBL- *E.coli* isolates.

The disc diffusion method was applied to all collected presumptive *E.coli* isolates to check the presence of organisms producing ESBL. For this purpose, antibiotic discs containing 30 μg each of cefotaxime, ceftriaxone, ceftazidime, aztreonam, and cefpodoxime were used. Isolates that tested resistant to these 3^rd^ generation cephalosporins were further analyzed to confirm the presence of ESBLs through phenotyping. In this study, *Escherichia coli* samples showing resistance to these 3^rd^ generation cephalosporins (ceftazidime, ceftriaxone, cefotaxime) were identified as possible ESBL producers and underwent additional test for phenotypic to validate the presence of ESBLs.

ESBL formation was validated via double disk synergy (DDST) as well as using combined disc tests (CDT) [10]. The assay utilized cephalosporin antibiotics, both alone and in conjunction with clavulanate. The bacterial cultures were incubated overnight at temperature of 35°C to 37°C. The presence of clavulanate, a compound that inhibits β-lactamase activity, led to the formation of a distinct keyhole or zone of enhancement, indicating positive for extended-spectrum beta-lactamases. Reference strains were utilized such as *E. coli* ATCC 35218 (β-lactamase-positive control for inhibitor tests) and ATCC 25922 (ESBL-negative control), while the *K. pneumoniae* ATCC 700603 was used as positive control for ESBL production. For the preservation of bacterial strains, brain heart infusion broth (BHI) containing glycerol (15%) was utilized for storage, maintained at −85°C until needed for further experimentation.

#### Serogrouping.

Serogrouping of confirmed *E. coli* isolates was performed using polyvalent antisera (Mast, United Kingdom), in accordance with the manufacturer’s protocol. These antisera containing three specific pools targeting different serogroups such as poly group 2, poly group 3 and poly group 4. Isolates that exhibited agglutination with the polyvalent antisera were further analyzed using monovalent O antisera for verification, ensuring accurate identification of serogroups. In addition to the Serogrouping process, controls for validation were used to ensure the reliability of the results. Positive controls, which were known isolates of *E. coli* with defined serogroups, were included in each assay to confirm the effectiveness of the antisera. Negative controls, such as non-*E. coli* isolates, were also essential to rule out cross-reactivity.

#### Antibiotic susceptibility Testing (AST).

The AST was conducted adhering to the standard agar disc diffusion method utilizing template discs (AB Biodisk, Solna, Sweden) on Mueller Hinton agar (MHA), strictly following Clinical and Laboratory Standards Institute (CLSI) M100 guidelines (2021). The isolates were tested against the following antibiotics with their respective standard disc potencies: amikacin (30 µg), amoxicillin/clavulanate (20/10 µg), ampicillin (10 µg), cefepime (30 µg), cefoperazone/sulbactam (75/30 µg), cefotaxime (30 µg), cefoxitin (30 µg), ceftazidime (30 µg), ceftriaxone (30 µg), ciprofloxacin (5 µg), imipenem (10 µg), meropenem (10 µg), piperacillin/tazobactam (100/10 µg), and sulfamethoxazole/trimethoprim (23.75/1.25 µg). Following 18–24 hours of incubation at 37°C, the zones of inhibition were measured. The results were interpreted by comparing the zone diameters to the interpretive criteria set forth in the CLSI 2021 guidelines [11].AST was performed to describe the in vitro susceptibility profiles of confirmed ESBL-producing *E.coli isolates..*

### PCR Screening for Selected ESBL-encoding genes

1. Bacterial Genomic DNA extraction was conducted by following the HiPur A DNA Purification kit (HIMEDIA) protocol.
2. DNA purity was assessed using a NanoDrop™ (Thermo Scientific, Waltham, Massachusetts, USA) to measure the optical density (OD) at A260 and A280. An A260/ A280 ratio ranging from these values (1.80–1.96), indicated that the DNA wasof good quality.
3. Primer: Specific primers targeting the ESBL responsible genes of interest (*bla*_TEM_, *bla*_SHV_, *bla*_CTX-M-group 1_, *bla*_CTX-M-group 9_, and *bla*_CTX-M-group 25_) were adopted from the literature (Table 1). The primer panel does not cover the full ESBL spectrum (e.g., CTX-M-2 group, CTX-M-8 group, or AmpC β-lactamases), which is a limitation of this study. PCR amplification was performed using the acquired primers with DNA target genes. PCR reaction mixtures (25 µL) contained 12.5 µL 2 × Master Mix, 1 µL each forward and reverse primer (10 µM), 2 µL DNA template, and 8.5 µL nuclease-free water. Amplification conditions were: initial denaturation at 94°C for 5 min; 35 cycles of 94°C for 30 s, annealing at the gene-specific temperature (Table 1) for 30 s, 72°C for 1 min; and final extension at 72°C for 10 min. Table 1 shows the different primers targeting the genes of interest:

**Table 1:** Primers targeting the ESBL- target genes.

Encoding Genes		Primer Sequence (5’-3’)	Annealed Temperature (◦C)	Expected size (bp)	Sources
**TEM**	Forward	TCGCCGCATACACTATTCTCA GAA TGA	**50**	445	[12]
	Reverse	ACGCTCACCGGCTCCAGATTT AT			
**SHV**	Forward	ATG CGT TATATT CGC CTG TG	**50**	747	[13]
	Reverse	TGC TTT GTT ATT CGG GCC AA			
**CTX-M-1**	Forward	ATGGTTAAAAAATCACTGCGTC	**54**	863	[14]
	Reverse	TTGGTGACGATTTTAGCCGC			
**CTX-M-9**	Forward	ATGGTGACAAAGAGAGTGCA	**54**	869	[14]
	Reverse	CCCTTCGGCGATGATTCTC			
**CTX-M-25**	Forward	CACACGAATTGAATGTTCAG	**54**	925	[15]
	Reverse	TCACTCCACATGGTGAGT			

The detection of target ESBL-encoding genes involved comparing the sizes of the PCR products with the markers present on the gel. The presence of genes is revealed by the specific bands exhibited (*bla*_TEM_, *bla*_SHV_, or *bla*_CTX-M_) for each sample. In every run, the positive control (ESBL-positive *E. coli*) and negative control (nuclease-free water) were employed [16]. After amplification, the PCR products underwent analysis via gel electrophoresis and visualized under ultra violet light after ethidium bromide staining. Sizes of the amplified DNA bands were verified to a MW marker, specifically the Gene Ruler of DNA ladder of 1 Kb Plus.

## Results

**Fig 1:**
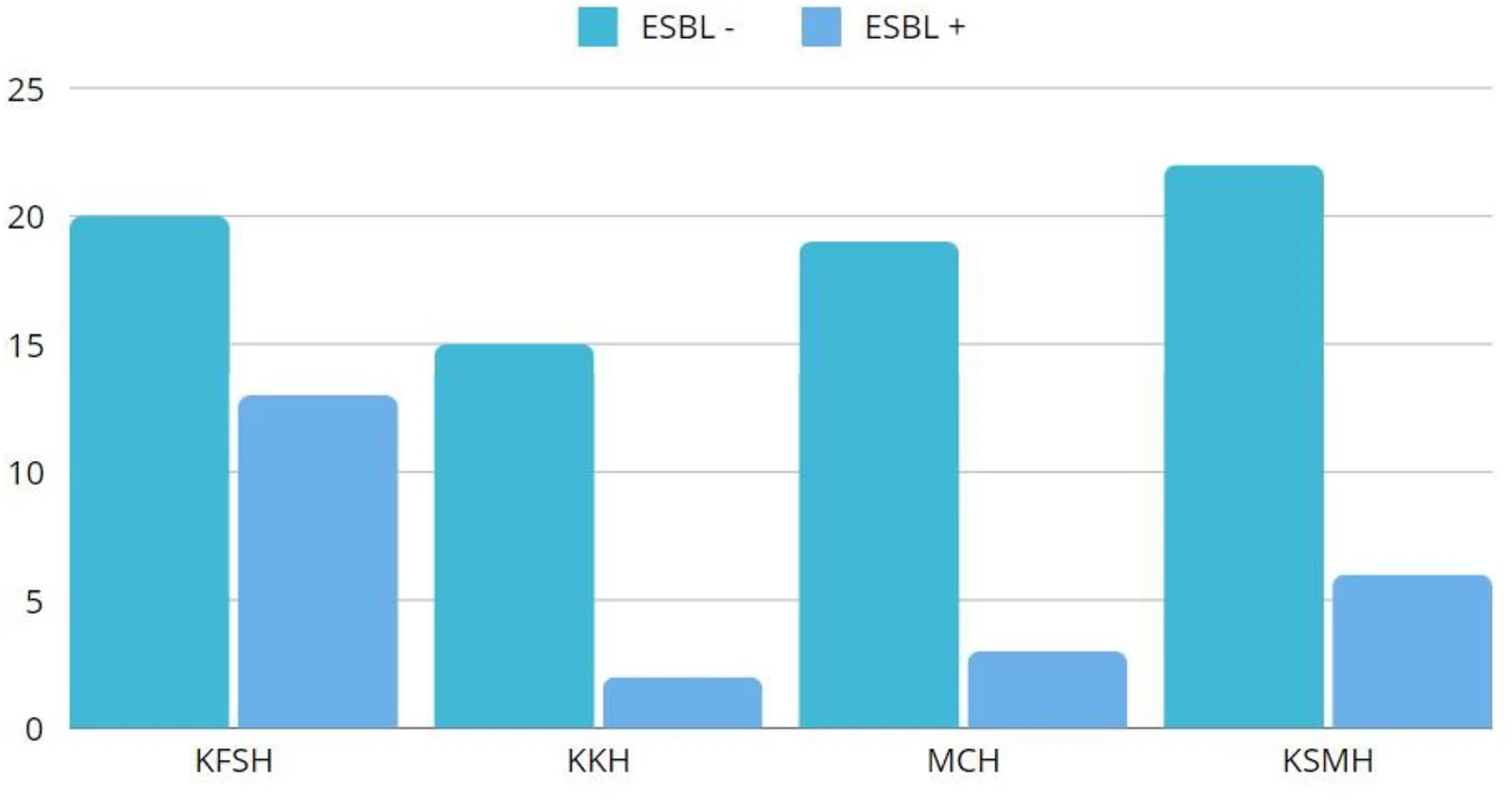
Percentage of *E.coli* isolates in the different hospitals.

Bacterial samples were obtained from different hospitals in Tabuk. A total of 100 *E.coli* isolates, comprising of (33) 33% came from King Fahad Specialist Hospital (KFSH) which has the largest number of samples obtained. While in King Khaled Hospital (KKH), there were 17 (17%) samples. About 22 (22%) from Maternity and Children Hospital (MCH), and 28(28%) from King Salman Hospital. A total of 24(24%) were ESBL positive and 76 (76%) were ESBL negative as shown in Fig 1.

**Fig 2:**
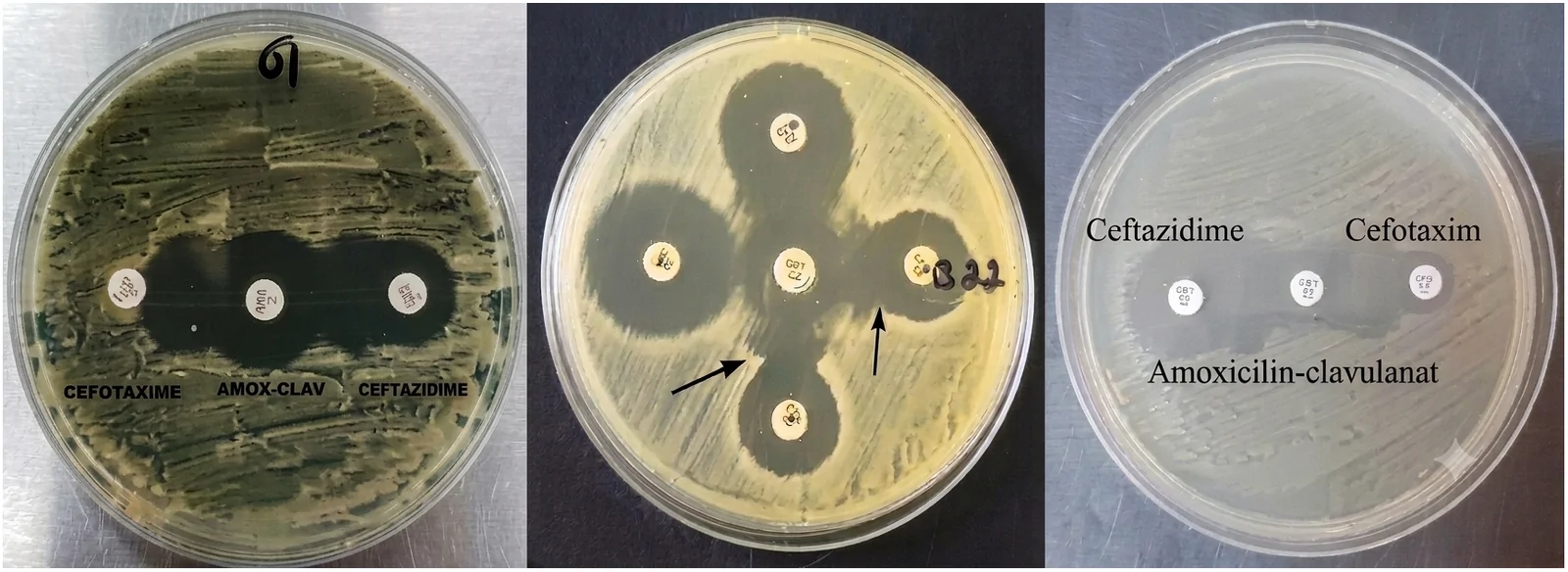
DDST result confirmed the ESBL-producing *E.coli* exhibiting synergy between the 3^rd^ generation cephalosporins and clavunate.

A total of 100 non-duplicate, consecutive clinical *E. coli* isolates were collected during the six-month study period from December 2023 to May 2024.. All clinical isolates (vary from urine, wound swab, and blood) were analyzed via Vitek system II for phenotypic identification and ESBL confirmatory. Only 24 clinical isolates were confirmed to be *E.coli positive for ESBL*. Most of ESBL isolates originated from urine samples (n = 17; 70.8%), while the remaining ESBL isolates were from blood (n = 4; 16.5%) and wound swabs (n = 3; 12.5%). The DDST result is shown in Fig 2. Demonstrating synergy (keyhole effect) between cephalosporins and clavulanate.

**Fig 3:**
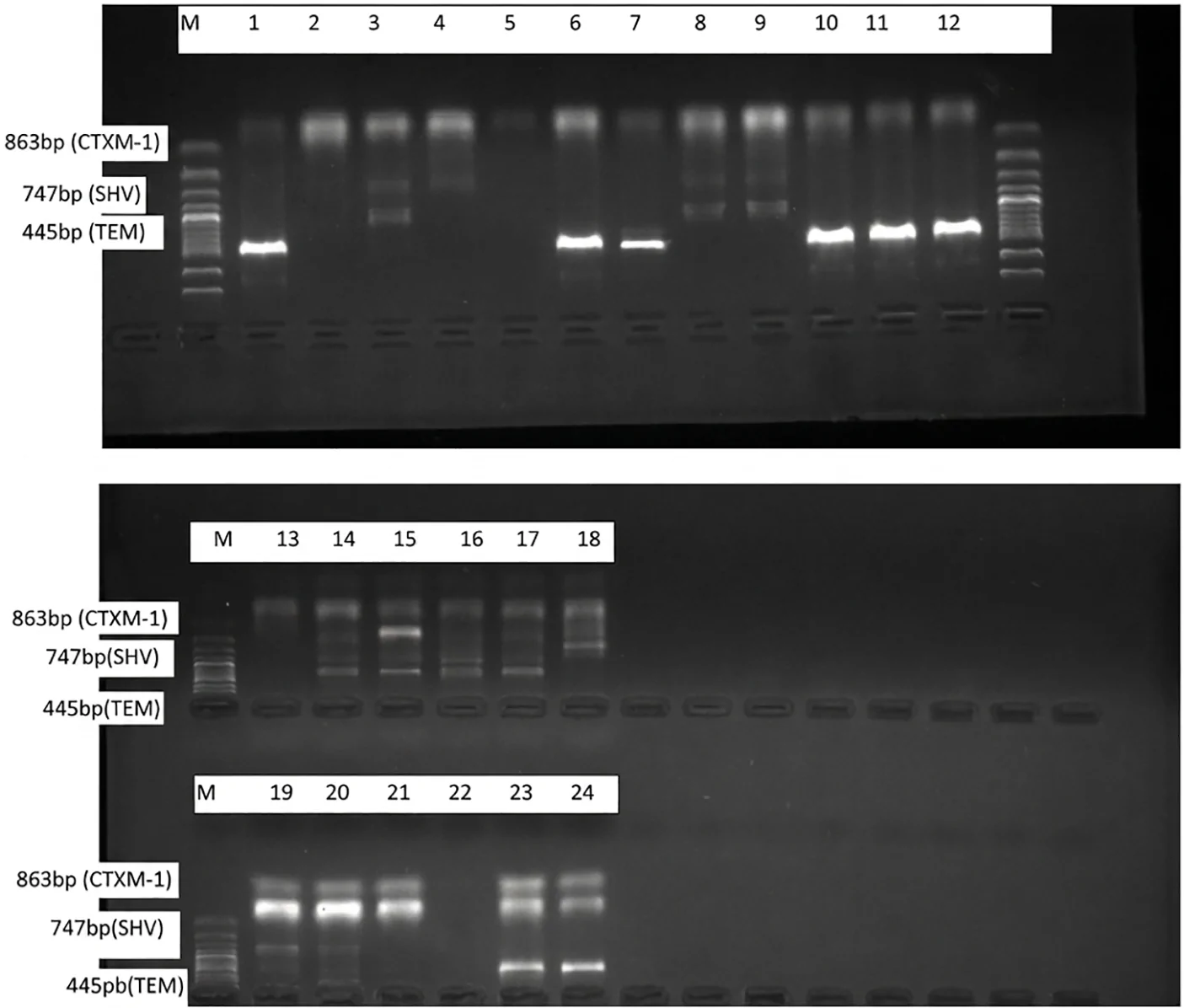
Gel electrophoresis results for the _*bla*_*CTX-M-group1*__, *bla*_*SHV*_ and *bla*_*TEM*_ in ESBL- *E. coli.*

**Fig 4:**
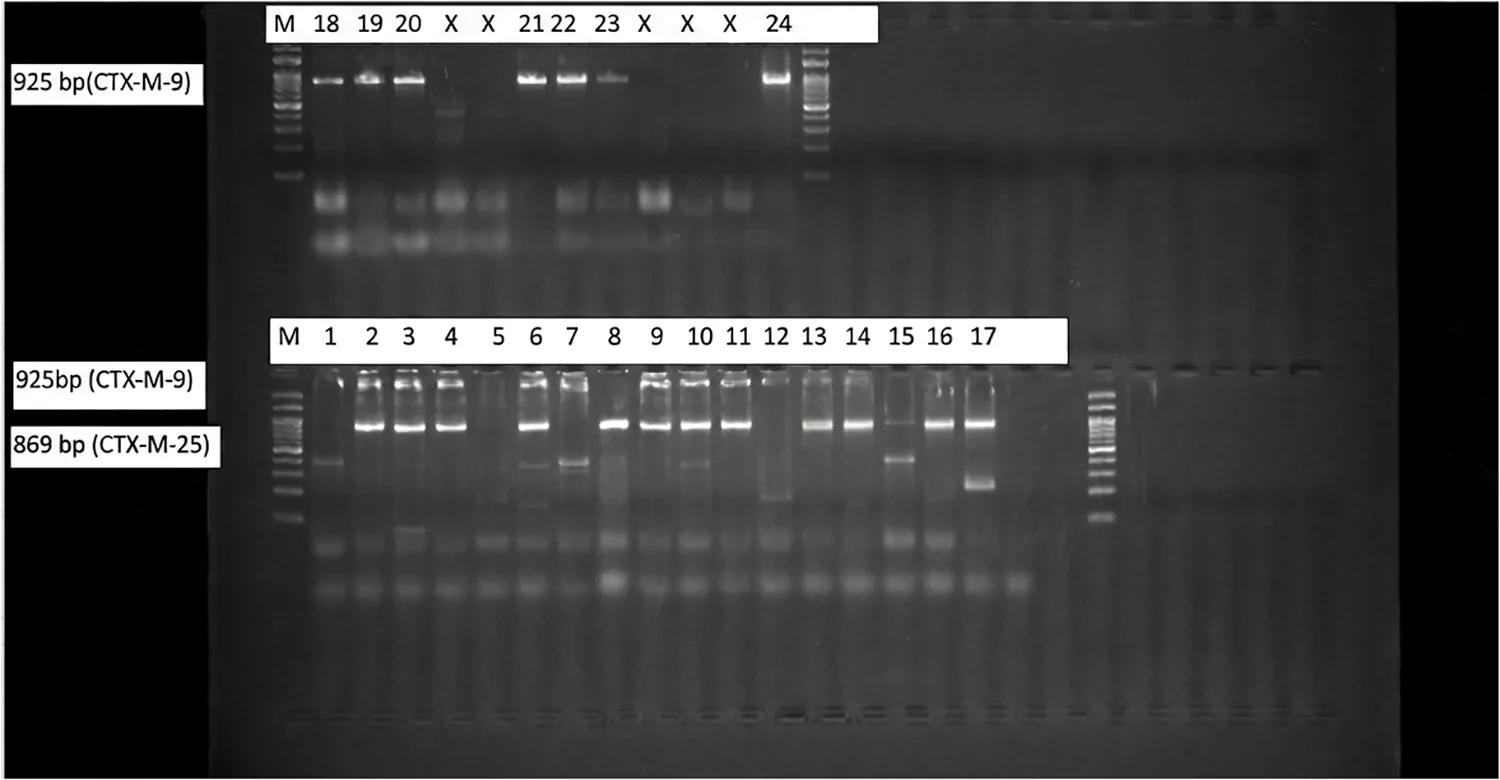
Gel electrophoresis results of the PCR products for *bla*_*CTX-M-25*_ and *bla*_*CTX-M- group 9*_ in ESBL- *E. coli.*

PCR amplification showed the occurrence of *bla*_TEM_, *bla*_SHV_, *bla*_CTX-M-group1_, *bla*_CTX-M-group9_, and *bla*_CTX-M-group25_ in 50%, 46%, 67%, 63%, and 50% of the 24 ESBL positive *E. coli* isolates, respectively as displayed in (Table 2 and Fig 3.) The *bla*_TEM_ and *bla*_CTX-M-group1_ co-occurred in 25% of isolates, while 10 (42%) of isolates co-harbored *bla*_SHV and_ *bla*_CTX-M-group1_. Three isolates (12.5%) harbored both *bla*_TEM_ and *bla*_SHV_ genes as shown in Table 2 and Fig 4. As shown in Table 2, these findings indicate that while certain resistance genes co-occur, their frequencies vary.

**Table 2:** Distribution in percentage of *bla*_SHV_, *bla*_TEM_, *bla*_CTX-M-1_, *bla*_CTX-M-9_ and *bla*_CTX-M-25_ in ESBL- *E.coli* samples.

ENCODING TARGET GENES	% distribution in ESBL-producing *E.coli*
TEM	12 (50%)
SHV	11 (46%)
CTX-M-1	16 (67%)
CTX-M-9	15 (63%)
CTX-M-25	12 (50%)
TEM, SHV	3(12.5%)
TEM, CTX-M-1	6 (25%)
SHV, CTX- M-1	10 (42%)
CTX M-9, CTX M-25	7 (29%)

Serotyping of these 24 ESBL positive *E. coli* isolates revealed that the predominant serogroups were O127 and O142, being presented by 8 (33.3%) and 5 (20.8%) isolates, respectively, followed by O86 (n = 6; 25%) and O111 (n = 3; 12.5%). However, two isolates (8%) were 2 non-typeable (ONT).

As shown in Table 3, the ESBL producers demonstrated complete resistance (n = 24, 100%) against ampicillin, cefotaxime, ceftazidime, and ceftriaxone. Among the remaining cephalosporins, cefoxitin exhibited a high resistance rate of 95.8%, with only 1 isolate (4.17%) classified as intermediate. Cefalotin was resistant in 15 isolates (62.5%), while 9 isolates (37.5%) showed intermediate results. Conversely, cefepime showed 6 isolates (25%) that are resistant, 3 isolates (12.5%) exhibiting intermediate susceptibility, and susceptible 15 isolates (62.5%).

**Table 3:** Resistant Patterns of Antimicrobial susceptibility in ESBL clinical isolates (*Escherichia coli*).

Family	Type	ESBL positive (n = 24)		
***Penicillin***	**Antibiotic**	**Resistant**	**Sensitive**	**Intermediate**
	**Ampicillin**	**24(100%)**	**0**	**0**
	**Amoxicillin/** **Clavulanate**	**15(62.5%)**	**3(12.5%)**	**6(25%)**
	**Piperacillin/** **Tazobactam**	**17(70.83%)**	**1(4.17%)**	**6(25%)**
***Cephalosporins***	**Cefalotin**	**15(62.5%)**	**0**	**9(37.5%)**
	**Cefoxitin**	**23(95.8%)**	**0**	**1(4.17%)**
	**Cefotaxime**	**24(100%)**	**0**	**0**
	**Ceftazidime**	**24(100%)**	**0**	**0**
	**Ceftriaxone**	**24(100%)**	**0**	**0**
	**Cefepime**	**6(25%)**	**3(12.5%)**	**15(62.5%)**
***Carbapenems***	**Imipenem**	**0**	**24(100%)**	**0**
	**Meropenem**	**0**	**24(100%)**	**0**
***Monobactam***	**Aztreonam**	**19(79.16%)**	**2(8.33%)**	**3(12.5%)**
***Aminoglycosides***	**Amikacin**	**0**	**18 (75%)**	**6(25%)**
***Fluoroquinolones***	**Ciprofloxacin**	**13(54.1%)**	**4(16.6%)**	**7 (29.16%)**
	**Ofloxacin**	**20 (83.3%)**	**0**	**4(16.6%)**

**N.B.** Antibiotic susceptibility testing was performed by the Kirby-Bauer disc diffusion method using discs of standard CLSI (2021) potencies: Ampicillin (10 µg), Amoxicillin/Clavulanate (20/10 µg), Piperacillin/Tazobactam (100/10 µg), Cefalotin (30 µg), Cefoxitin (30 µg), Cefotaxime (30 µg), Ceftazidime (30 µg), Ceftriaxone (30 µg), Cefepime (30 µg), Imipenem (10 µg), Meropenem (10 µg), Aztreonam (30 µg), Amikacin (30 µg), Ciprofloxacin (5 µg), Ofloxacin (5 µg).

In evaluating carbapenems and other antibiotics against the *E. coli* isolates, all these 24 isolates exhibited susceptibility to imipenem and meropenem (100%) Amikacin resistance was observed in 0 isolates (0%). However, for ciprofloxacin, 4 isolates (16.6%) were classified as sensitive, while 13 isolates (54.1%) were resistant. For ofloxacin, only 4 isolates (16.6%) were intermediate, and 20 isolates (83.3%) were resistant.

## Discussion

The assessment of antibiotic susceptibility in *E. coli isolates, positive to* these Extended-Spectrum Beta-Lactamase (ESBL) enzymes highlights the significant variation in effectiveness across different antibiotic classes. Carbapenems, specifically Imipenem and Meropenem, exhibited complete efficacy, with 100% susceptibility, making them the most reliable treatment choices in vitro for infections caused by these resistant strains. A study conducted by Abdulaziz Alqasim and colleagues in Riyadh, Saudi Arabia, reported a troubling prevalence of multi-resistance to these antibiotics among uropathogenic *E. coli*, with 67% of these isolates showing multidrug resistance and ESBL producers being resistant to various antibiotics. The findings emphasized the effectiveness of carbapenems, such as imipenem, due to their high sensitivity in *E. coli* isolates [17].

The study demonstrates that ESBL producers showed complete resistance (n = 24, 100%) against ampicillin, cefotaxime, ceftazidime, and ceftriaxone. This aligns with previous research indicating a concerning trend in ESBL-producing *E. coli* strains, which often exhibit high resistance to these commonly used antibiotics [18].

Conversely, cefepime demonstrated a lower resistance rate, with only 6 isolates (25%) resistant and 3 isolates (12.5%) exhibiting intermediate susceptibility, alongside a significant susceptibility rate of 15 isolates (62.5%). This indicates that cefepime may remain a viable treatment option, especially for infections stemming from specific strains of ESBL- *E. coli*. Notably, some studies described that cefepime can be effective against CTX-M ESBL- *E. coli* within specific minimum inhibitory concentration ranges [19]. The finding that only one out of 24 ESBL-producing *E.coli* isolates (4.17%) remained susceptible to piperacillin/tazobactam (TZP) stands in contrast to figures commonly reported from other studies within Saudi Arabia and neighboring regions, where susceptibility rates for this combination typically fall between 40% and 70% [20,21]. This study adhered to the CLSI M100 2021 standards, which introduced a Susceptible-Dose Dependent (SDD) category specifically for TZP. The disc diffusion method employed in this study does not allow assignment of the SDD category; isolates that would be classified as SDD by broth microdilution are often reported as intermediate or resistant based solely on zone diameter thresholds. As a result, some isolates in this study that might be treatable with aggressive TZP dosing could have been incorrectly categorized as non-susceptible. The combination of a high carriage rate of *bla*_CTX-M-1_ (67%) together with frequent co-occurrence of *bla*_TEM_ (50%) and *bla*_SHV_ (46%) within individual isolates may produce a cumulative hydrolytic effect against TZP that exceeds what is typically observed with single ESBL enzymes. Additionally, the unusually high resistance rate to cefoxitin (95.8%) raises the possibility that many of these isolates co-produce AmpC β-lactamases or harbor porin deficiencies, both of which are known to diminish TZP activity independently of ESBL production. This study did not perform phenotypic confirmation of AmpC production, representing an acknowledged limitation. Moreover, comparisons with earlier regional studies must be made cautiously, as those investigations often employed older CLSI breakpoints that predated the SDD distinction or were conducted in different hospital environments with unique antimicrobial prescribing practices. Future research in the Tabuk region should incorporate MIC-based testing for TZP to enable proper SDD categorization, alongside targeted testing for AmpC enzymes and porin alterations.

The near-complete resistance to cefoxitin (95.8%) observed in this study strongly suggests possible co-production of AmpC β-lactamases, a phenomenon reported in other studies from the region as a potential driver of treatment failure with cephalosporin/β-lactamase inhibitor combinations. This finding warrants further investigation, as phenotypic confirmation of AmpC production was not performed in the current study.

The occurrence of *bla*_CTX-M_ group 1 type 15 among gram-negative bacilli isolates, which showed no resistance to imipenem and meropenem, suggests these antibiotics may be effective against *bla*_CTX-M_-carrying strains [22]. Long-term treatment approaches for ESBL-producing E. coli infections have also been explored, including the use of alternative agents such as pivmecillinam [23]. Current strategies for treating infections caused by *bla*_TEM_-carrying bacteria often involve carbapenems, which are effective against many resistant strains [24]. Additionally, ongoing research is exploring gene therapy as a novel approach to address antibiotic resistance, particularly targeting genes like *bla*_TEM_ [25]. Collectively, these results and related studies highlight the global challenge of MDR ESBL-producer *E. coli* and may provide insights into possible treatment strategies. Routine susceptibility testing remains crucial for guiding effective treatment.

The study of encoding genes associated with ESBL- production among *E. coli* isolates in Tabuk hospitals reveals significant perspectives into the prevalence of various multi-resistance genes in the Tabuk, Saudi Arabia. This *bla*_TEM_ gene present and detected among the 12 isolates, accounting for 50% of the total, indicating a significant presence of this gene, which yields resistance to these variety of beta-lactam antibiotics. The *bla*_SHV_ gene was found in 11 isolates, representing 46%, suggesting a moderate prevalence of this resistance mechanism. The *bla*_CTX-M-1_ gene detected among 16 isolates (67%), highlighting it as the gene with the highest prevalence. The *bla*_CTX-M_ family, particularly *bla*_CTX-M-1_, is associated with significant resistance to cephalosporins, raising concerns in clinical settings.

The gene (*bla*_CTX-M-9_), detected in 15 isolates (63%), while *bla*_CTX-M-25_ gene was identified from the 12 isolates (50%). Both genes contribute to cephalosporin resistance, reflecting their relevance in the resistance landscape of ESBL- *E. coli*. Three isolates (12.5%) harbored both the *bla*_TEM_ and *bla*_SHV_ genes, The combination of these *bla*_TEM_ and *bla*_CTX-M-1_ was detected in 6 isolates (25%), and the *bla*_SHV_ and *bla*_CTX-M-1_ combination was in 10 isolates (42%), indicating multiple resistance mechanisms within certain isolates.

The observation that *bla*_TEM_ and *bla*_SHV_ co-occurred in only 12.5% of isolates, while *bla*_CTX-M-1_ co-occurred more frequently with other genes, may reflect the distribution of resistance determinants in this sample. Possible explanations include the small sample size (n = 24), local clonal expansion, plasmid incompatibility, or stochastic distribution. The study design (cross-sectional, descriptive) does not permit inferences about competitive advantage, selective pressure, or evolutionary dynamics. [26].

The predominance of *bla*_CTX-M-group1_, (67% of isolates) highlights its role as a common resistance determinant in this population. The distinct patterns of resistance gene distribution emphasize the necessity for ongoing surveillance and a deeper comprehension of the process behind antibiotic resistance of *E.coli*. This is particularly relevant in light of studies that have shown that the presence of certain ESBL genes may be associated with the absence of others due to various factors, including plasmid characteristics [27,28].

Furthermore, the dynamics of resistance gene co-occurrence can influence treatment outcomes and public health strategies, as the presence of multiple resistance genes often correlates with increased virulence and treatment failure [29]. Understanding these interactions is crucial for developing effective interventions against multidrug-resistant *E. coli* strains.

The investigation of *E. coli* isolates showed that the highest prevalence in serogroups was O127 and O142. The clinical implications of identifying specific serogroups of *E. coli* are significant, particularly in relation to their association with extended-spectrum beta-lactamases (ESBLs). Certain serogroups, such as O127 and O86, have been linked to higher virulence and increased resistance to antibiotics in some studies [30]. Notably, serogroup O127 is traditionally categorized among enteropathogenic *E.coli* (EPEC), which are significant causes of infantile diarrhea. The finding of this serogroup in urine samples from adult patients in Tabuk raises questions about the potential environmental dissemination and adaptation of these strains in the region.

Moreover, the existence of ESBL-positive strains within specific serogroups may contribute to outbreaks of multidrug-resistant infections. Studies have shown that serogroups like O111 and O142 are often implicated in nosocomial infections, where ESBL production further complicates antibiotic therapy [31]. The diversity of serogroups reported here (O127, O142, O86, O111) suggests a complex epidemiological reservoir in Tabuk. Tabuk’s unique geography as a hub for regional trade and travel may influence its antimicrobial resistance patterns, though this study did not directly investigate transmission routes. The serogroup findings from clinical isolates of *E. coli* not only provide insights into the epidemiology of infections but also underscore the relationship between specific serogroups and the prevalence of ESBLs, that has critical impact for clinical management [32,33].

The proliferation of drug resistance in Gram-negative organisms, including ESBL-producing *E. coli*, presents notable public health concerns. This issue is primarily due to the unique outer membrane of these bacteria, which acts as a barrier against many antibiotics, reducing their efficacy. In Saudi Arabia, the presence of drug resistance is particularly concerning, with studies indicating high rates among common pathogens, especially in healthcare settings where improper usage of antibiotics has fuelled the emergence of drug resistance. The prevalence of ESBL-positive *E. coli* in Saudi Arabia has been reported to exceed 50% in some cases [34]. Numerous studies have emphasized the existence and spread of β-lactamase-resistant organisms in the country. Al-Hasan et al. [35] documented the presence of ESBL-positive Enterobacteriaceae in hospitals nationwide, indicating a widespread distribution of ESBL genes.

Molecular epidemiology studies have extensively characterized the antibiotic susceptibility and common genetic mechanisms of ESBL-positive *E. coli* in various locations, revealing a concerning trend of multidrug resistance (MDR) with their distribution of ESBL genes [6]. For instance, research conducted at King Abdullah Hospital indicated high rates of MDR among bacterial species, with the *bla*_TEM_ gene showing the highest prevalence [8]. Similarly, studies from Pakistan have shown that third-generation cephalosporins were significantly resistant, with *bla*_CTX-M_ and *bla*_TEM_ genes common among the isolates [36]. In Sudan, a notable prevalence of ESBL production and the occurrence of *bla*_TEM_, *bla*_CTX-M_, and *bla*_SHV_ genotypes, particularly in *K. pneumoniae and E. coli*, has been noted.

Research conducted in Eastern Saudi Arabia identified similar important prevalence of ESBL-positive *E. coli* associated with urinary tract infections, and the *bla*_CTX-M_ gene was identified frequently [20]. Furthermore, studies from South Africa using Multiplex-PCR assays found an 87% prevalent rate of ESBL (+) genes among clinical isolates, the CTX-M group 1 being the most prevalent [21]. The potential for Saudi Arabia to be a hotspot in transmission of cephalosporin-resistant pathogens, including CTX-M-producing strains, is heightened by international pilgrimages and regional population movements [37].

The findings from this study indicate that 24% of the clinical samples analyzed from Tabuk hospitals were ESBL positive, underscoring the extent of antibiotic resistance in the Tabuk Region. This prevalence underscores the need for robust infection control programs and antibiotic management plans The dominance of ESBL-producing *E. coli* among urinary samples aligns with global trends, as the infections in urinary tract (UTIs) have been common sources of these resistant isolates. The presence of these pathogens in blood and wound swab samples also indicates the potential for severe systemic infections, warranting careful monitoring and management.

The utilization of multiplex PCR has been validated to be an effectual method for the rapid identification of ESBL-associated genes. The widespread distribution of *bla*_TEM_ genes in *E. coli* strains aligns with previous reports indicating that these genes are among the most prevalent ESBL genes worldwide. Furthermore, identification of *bla*_CTX-M_ genes in most isolates is noteworthy, as CTX-M enzymes are recognized as significant contributors to resistance against extended-spectrum cephalosporins.

The results of this research corroborate previous studies regarding the presence of drug resistance among Gram-negative pathogens, particularly *E.coli*. The World Health Organization (WHO) [9] highlighted this growing concern regarding antibiotic resistance in these bacteria, and the current study provides further evidence of this issue, with 24% of the samples testing positive for ESBL production. Also, the study adds to the literature how these dissemination of beta-lactamase resistance genes occurred especially in Tabuk region. Shafiq et al. [4] discussed the impact of mobile genetic system on the spread of antibiotic resistance through different bacterial species, and this research supports that notion by detecting specific ESBL-responsible genes, such as *bla*_TEM_ and *bla*_CTX-M-1_, among these samples.

## Conclusion

The occurrence of positive ESBL-*E.coli* poses notable public health risks, particularly in Saudi Arabia. This study highlights the critical role of routine antibiotic susceptibility testing in characterizing resistance profiles of these multidrug-resistant strains in vitro. Notably, carbapenems—specifically imipenem and meropenem—demonstrated complete susceptibility(100%)among the tested isolates in this study. [17].

These findings reveal a concerning landscape of antibiotic resistance. While cefotaxime, ceftazidime, and ceftriaxone showed complete resistance, cefepime exhibited a susceptibility rate of 62.5%. This variability underscores the necessity for susceptibility testing to guide antibiotic selection. The predominance of the *bla*_CTX-M-1_ gene (67%) among isolates indicates this as a common genetic factor associated with cephalosporin resistance in this sample, as evidenced by previous research [38].

Given the presence of multiple resistance genes (*bla*_TEM_, *bla*_SHV_, and *bla*_CTX-M_ variants), routine antimicrobial susceptibility testing is recommended before treatment when feasible. This research highlights the presence of CTX-M-1 in ESBL-positive *E. coli* within the Tabuk region, offering information on local antibiotic resistance patterns.

Given the increasing challenge of antibiotic resistance, the findings of this research offer insights into the genetic epidemiology of *E. coli* strains harboring ESBL enzymes in the Tabuk region. Continued surveillance, susceptibility testing, and further molecular characterization (including sequencing and plasmid analysis) are needed to better understand resistance mechanisms in this setting. This study highlights the urgent need for comprehensive approaches to monitor antibiotic resistance in the Tabuk region and across Saudi Arabia as the worldwide burden of drug resistance continues to rise.

## Supporting information

S1 File S1_raw_images. (DOCX)
