# Supplementary material for: Distribution of blaCTX-M, blaTEM and blaSHV encoding-genes among extended-spectrum beta-lactamase (ESBL)-producing Escherichia coli isolates at the different hospitals in Tabuk, Kingdom of Saudi Arabia
Source: PLoS One. 2026 Sep 10;21(9):e0350207. doi: 10.1371/journal.pone.0350207 (PMC13561370; doi:10.1371/journal.pone.0350207)
Supplement: Supplementary file 1 [file pone.0350207.s001.docx]

Figure 3 Gel electrophoresis of the PCR products for bla CTX-M1, bla SHV and

bla TEM in ESBL- *Escherichia coli*

Figure 4. Gel electrophoresis of the PCR products for bla CTX-M-25 and bla CTX-M-9 in ESBL- *Escherichia coli*
